# Lipid Raft Association Stabilizes VEGF Receptor 2 in Endothelial Cells

**DOI:** 10.3390/ijms22020798

**Published:** 2021-01-14

**Authors:** Ibukunoluwapo O. Zabroski, Matthew A. Nugent

**Affiliations:** Department of Biological Sciences, University of Massachusetts Lowell, Lowell, MA 01854, USA; ibukunoluwapo_zabroski@student.uml.edu

**Keywords:** angiogenesis, cholesterol, endothelial cell, lipid rafts, lysosome, receptor, simvastatin, vascular endothelial growth factor

## Abstract

The binding of vascular endothelial growth factor A (VEGF) to VEGF receptor-2 (VEGFR-2) stimulates angiogenic signaling. Lipid rafts are cholesterol-dense regions of the plasma membrane that serve as an organizational platform for biomolecules. Although VEGFR2 has been shown to colocalize with lipid rafts to regulate its activation, the effect of lipid rafts on non-activated VEGFR2 has not been explored. Here, we characterized the involvement of lipid rafts in modulating the stability of non-activated VEGFR2 in endothelial cells using raft disrupting agents: methyl-β-cyclodextrin, sphingomyelinase and simvastatin. Disrupting lipid rafts selectively decreased the levels of non-activated VEGFR2 as a result of increased lysosomal degradation. The decreased expression of VEGFR2 translated to reduced VEGF-activation of the extracellular signal-regulated protein kinases (ERK). Overall, our results indicate that lipid rafts stabilize VEGFR2 and its associated signal transduction activities required for angiogenesis. Thus, modulation of lipid rafts may provide a means to regulate the sensitivity of endothelial cells to VEGF stimulation. Indeed, the ability of simvastatin to down regulate VEGFR2 and inhibit VEGF activity suggest a potential mechanism underlying the observation that this drug improves outcomes in the treatment of certain cancers.

## 1. Introduction

New blood vessels are formed from pre-existing ones in a tightly regulated, organized and sequential process known as angiogenesis. The improper regulation of angiogenesis can result in diseases such as wet age related macular degeneration, formation of leaky vasculature, cancers and embryonic lethality [1].

Vascular endothelial growth factor A isoform 165 (VEGF) is a signaling protein that has an affinity for specific receptor tyrosine kinase receptors on the endothelial cell surface [2]. The major receptors to which VEGF binds are vascular endothelial growth factor receptors 1 (VEGFR1) and 2 (VEGFR2). VEGFR1 has an impaired kinase activity such that the binding of VEGF appears not to induce an angiogenic effect, instead it is thought to function as a “decoy receptor” that represses VEGF-mediated angiogenic activity. VEGF binding to VEGFR2, which has a fully functional tyrosine kinase domain, activates ERK and various signaling cascades that lead to endothelial cell permeability, survival, migration and proliferation, all of which are required for angiogenesis [3,4]. VEGFR2 exists in 3 major forms: as a ~150 kDa unglycosylated molecule, ~200 kDa partially glycosylated intermediate molecule and a ~230 kDa fully glycosylated mature molecule [5,6].

Lipid rafts are ordered and tightly packed microdomains in the cell membrane that are rich in cholesterol, sphingolipids, saturated phospholipids, and glycosphingolipids. They are 50–100 nm in diameter and their insolubility in cold, non-ionic detergents such as 1% Triton X-100, has led them to be referred to as detergent resistant membranes [7,8]. Although lipid rafts are generally consistent in their lipid composition, they vary in density and the types of proteins that associate with them [9]. The glycosphingolipid GM-1 (monosialotetrahexosylganglioside) is consistently found in all lipid rafts and is used as a biomarker, whereas caveolin and flotillin are selectively present in a subset of rafts [10]. Some angiogenic proteins that have been shown to associate with lipid rafts include: mitogen activated protein kinases, Src family kinases, endothelial nitric oxide synthase, protein kinase A, protein kinase C, and receptor tyrosine kinases [7,8,11]. Overall, lipid rafts are transient, heterogenous in nature, and serve as an organizational framework for receptors and associated signaling molecules [12,13].

Altering cellular cholesterol levels by sequestration, depletion and inhibition its of biosynthesis, and degradation of sphingomyelin are common tools used to disrupt lipid rafts with the aim of studying their functions [7,14]. Although initially discovered for use in the management of hypercholesterolemia, HMG-CoA reductase inhibitors, generally referred to as statins, have been shown to exhibit pleiotropic effects and, thus, find use in immunomodulation, plaque stabilization, anti-oxidation, and surprisingly, angiogenesis [15,16]. In several clinical studies, simvastatin, either alone or in combination with a chemotherapeutic, has been shown to improve therapeutic outcomes and decrease mortality in patients with breast, prostate, lung and colorectal cancers [17,18].

It has been hypothesized that the stability of lipid rafts plays an important role in ligand induced activation of associated receptors and recruitment of signal transduction molecules. For example, in endothelial cells, VEGFR2 has been shown to colocalize with lipid rafts to regulate VEGF induced signal transductions that are necessary for angiogenesis [13]. Disrupting lipid rafts in bovine aortic endothelial cells (BAECs), spontaneously immortalized human umbilical vein endothelial cell line (ECV304) and human acute myeloid leukemia cells (B1647) increases both basal phosphorylation levels and VEGF mediated phosphorylation of VEGFR2 [13,19,20]. Most previous studies have focused on activated VEGFR2, ligand mediated translocation of VEGFR2 and downstream effectors in and out of lipid rafts.

Proteolysis of VEGFR2 involves a coordinated interaction between the acidic lysosome and the more selective proteasome [21,22]. In certain cases, there is a redirection of VEGFR2 targeted for degradation between the two proteolytic pathways. For example, inhibiting the tyrosine kinase domain of VEGFR2 and de-ubiquination of VEGF activated VEGFR2 redirects the VEGF-VEGFR2 complex for lysosomal degradation [23,24]. VEGFR2, when activated by VEGF, undergoes receptor mediated endocytosis to induce signal transduction, and eventually targets VEGFR2 for lysosomal degradation [21,25,26,27]. Independent of VEGF, VEGFR2 has been shown to undergo constitutive endocytosis into recycling vesicles with 60% present at the cell surface at any given time [26,27]. This ligand-independent and clathrin-dependent constitutive endocytosis was found to protect VEGFR2 from ectodomain proteolysis [5].

Aside from its colocalization with lipid rafts, little is known about how raft localization impacts non-activated VEGFR2. In particular, the possibility that raft association of non-activated VEGFR2 modulates its homeostasis, and in turn, cellular responsiveness to VEGF has not been explored in detail. In the present study, we evaluated the effect of lipid rafts on non-activated VEGFR2 in endothelial cells in vitro using a combination of cholesterol modulating agents, simvastatin and methyl-β-cyclodextrin (MβCD), and sphingomyelinase (SMase), an enzyme that degrades sphingomyelin in lipid rafts. We found that all three agents led to down regulation of steady state VEGFR2 levels and selectively increased the lysosomal degradation of non-activated VEGFR2, which consequently reduced VEGF induced ERK activation. This study suggests that lipid rafts regulate non-activated VEGFR2 levels and in turn, modulate the cellular response to VEGF. It also provides a potential molecular mechanism underlying the successful use of simvastatin in the management of certain cancers and suggest other anti-VEGF applications for this common drug.

## 2. Results

### 2.1. Disruption of Lipid Rafts in Endothelial Cells Selectively Decreases the Levels of VEGFR2

**Figure 1:**
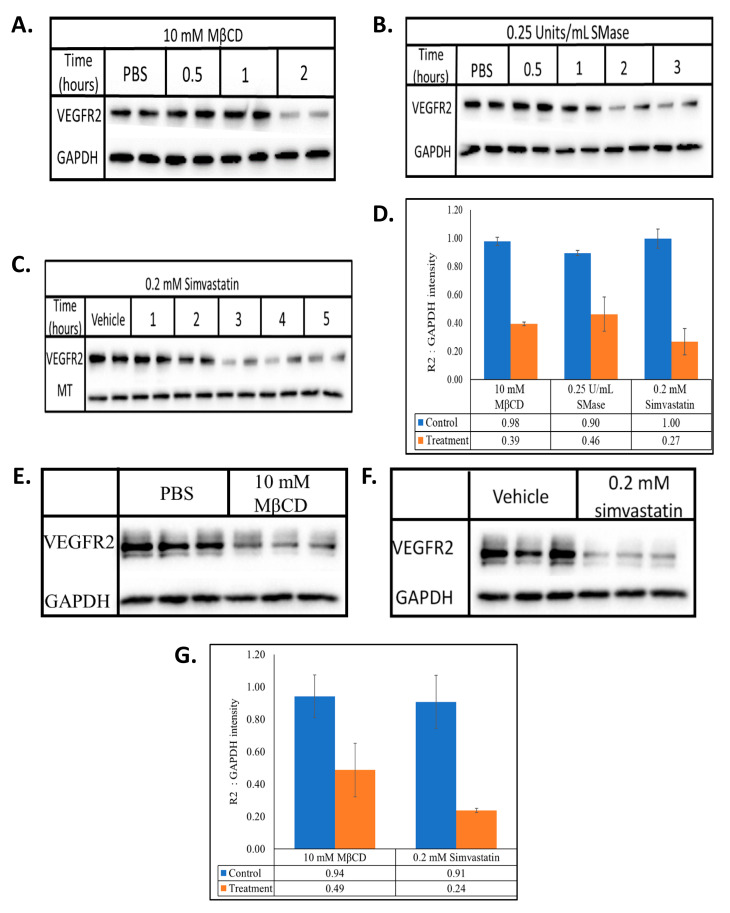
MβCD, SMase and simvastatin reduces the steady state levels of VEGFR2. BAECs were treated with (**A**) 10 mM MβCD, (**B**) 0.25 U/mL SMase, or (**C**) 0.2 mM simvastatin for the indicated times. HUVECs were treated with (**E**) 10 mM MβCD for 1 h, or (**F**) 0.2 mM simvastatin for 2 h. Whole cell lysates were collected, normalized for total protein and subjected to SDS-PAGE and Western blotting. All blots were densitometrically quantified by ImageJ, VEGFR2 band density was normalized to the respective housekeeping proteins (GAPDH or MT) and the average ±SD represented in the graphs (**D**,**G**).

The heterogeneity of the structural components that make up lipid rafts allow for their disruption in different ways. MβCD is a chemical that causes the efflux of cellular membrane cholesterol, SMase is an enzyme that degrades sphingomyelin and simvastatin is an inhibitor of HMG-CoA (3-hydroxy-3-methylglutaryl-CoA) reductase leading to inhibition of the biosynthesis of cellular cholesterol [28,29,30]. Two hour treatment with MβCD, 2 h treatment with SMase and 3-h treatment with simvastatin reduced VEGFR2 levels in BAECs by ~60, 50 and 70%, respectively (Figure 1A–C). Although all three agents target lipid rafts through distinct mechanisms, their outcome on the steady state levels of VEGFR2 in BAECs was similar. To explore if this alteration in VEGFR2 expression levels was peculiar to BAECs, the lipid rafts of HUVECs were disrupted in a similar manner. MβCD and simvastatin reduced VEGFR2 levels by ~50 and 70%, respectively (Figure 1E–G). These results suggest that lipid raft-modulation of VEGFR2 homeostasis is likely reflective of a general process by which lipid rafts are able to regulate VEGFR2 levels in endothelial cells and not one that is unique to the BAEC system. Thus, for the remainder of the study we utilized the BAEC culture system.

**Figure 2:**
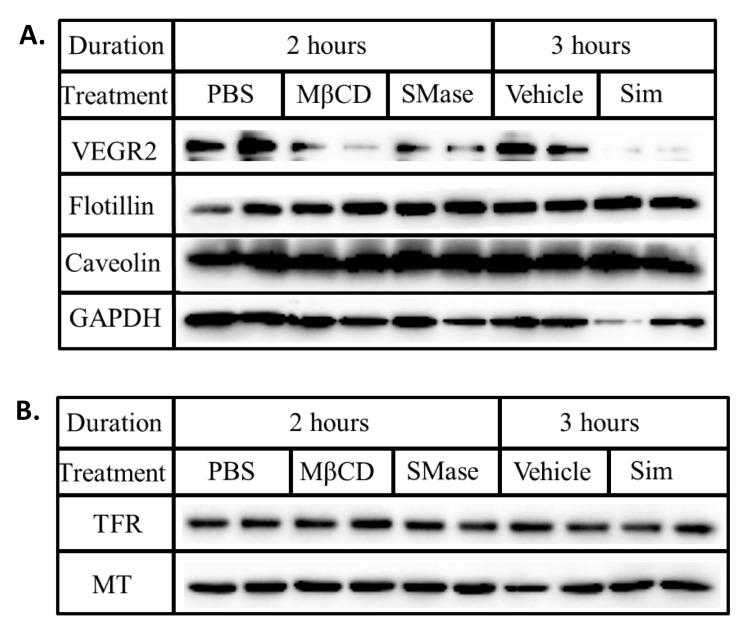
Lipid rafts disruption does not alter the steady state levels of caveolin, flotillin and TFR. BAECs were treated with ether MβCD (10 mM), SMase (0.25 U/mL) or simvastatin (0.2 mM) for the stated duration. Whole cell lysates were collected, normalized for total protein and subjected to SDS-PAGE and Western blotting. The presence of (**A**) VEGFR2, flotillin, caveolin, GAPDH, (**B**) TFR and MT were probed for using the respective antibodies.

To evaluate if the reduced levels of VEGFR2 associated with disrupting lipid rafts was a general phenomenon, we quantified the steady state levels of raft (caveolin and flotillin) and non-raft (TFR) associated proteins. Although disrupting lipid rafts with MβCD, SMase and simvastatin markedly lowered VEGFR2 expression, the levels of flotillin, caveolin (Figure 2A) and TFR (Figure 2B) remained unchanged. The fact that these proteins were unaffected suggests that disrupting lipid rafts may specifically influence membrane bound receptors such as VEGFR2 that associate with them.

### 2.2. MβCD, SMase, and Simvastatin Disrupt Lipid Rafts through Distinct Mechanisms

**Figure 3:**
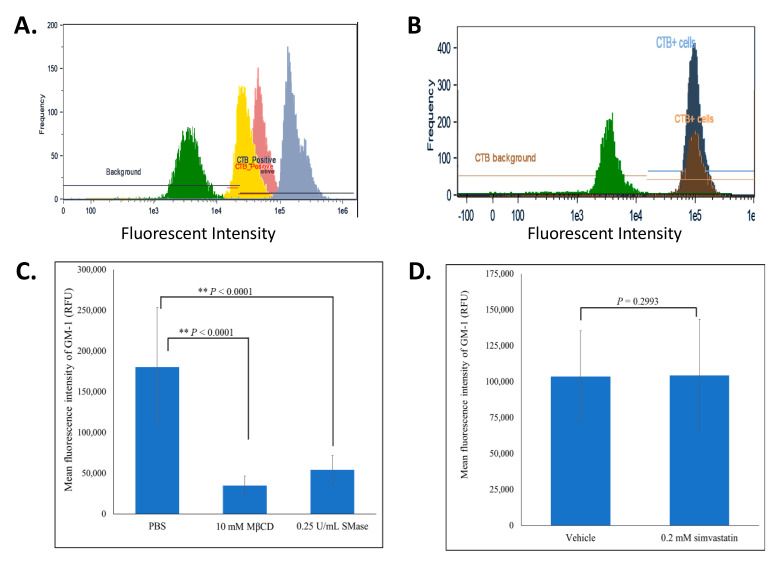
MβCD and SMase, but not simvastatin, reduced the levels of CTB-FITC bound GM-1. BAECs subjected to lipid rafts disruption were fixed, permeabilized and fluorescently labeled with CTB-FITC. During image flow cytometry, approximately 5000 cells were analyzed and a histogram of the frequency versus the intensity of CTB-FITC was generated. (**A**) represents an overlay of 4 different histograms representing the background fluorescence of a FITC-isotype control labeled cell population (green), untreated cells (blue), MβCD (10 mM; 2 h) treated cells (yellow), SMase (0.25 U/mL; 2 h) treated cells (pink). (**B**) represents an overlay of 3 different histograms representing the background fluorescence of a FITC-isotype control labeled cell population (green), vehicle (dark blue) and simvastatin (0.2 mM; 2 h) treated cells (brown). (**C**,**D**) represent the average mean fluorescent intensities ±SD for the samples resented in panels A and B, respectively. The two-tailed t-test was done and statistically significant conditions with *p*-values < 0.001 are indicated (**).

To further understand how MβCD, SMase and simvastatin impact lipid rafts, we fluorescently quantified the levels of CTB-FITC bound to GM-1 glycosphingolipid, a non-protein marker of lipid rafts, using image flow cytometry [31]. When compared to untreated cells, MβCD and SMase caused a marked reduction in the mean and maximum relative fluorescent unit (RFU) intensities of CTB-FITC bound GM-1 (Figure 3A and Table 1). Further analysis showed a significant reduction in the mean RFU by ~81 and 70% in cell populations treated with MβCD and SMase, respectively (Figure 3C and Table 1). These findings suggest that effluxing of membrane cholesterol and enzymatic degradation of sphingomyelin affects the stability of rafts associated GM-1. Surprisingly, simvastatin had no effect on the levels of GM-1 (Figure 3B,C and Table 1). The transient nature of lipid rafts may be evident here such that simvastatin may impact a minor sub-class of rafts or may interfere with the formation of new rafts rather than with existing ones. MβCD and SMase, however, may impact lipid rafts more uniformly, leading to the observed decrease levels of GM-1 gangliosides.

**Figure 4:**
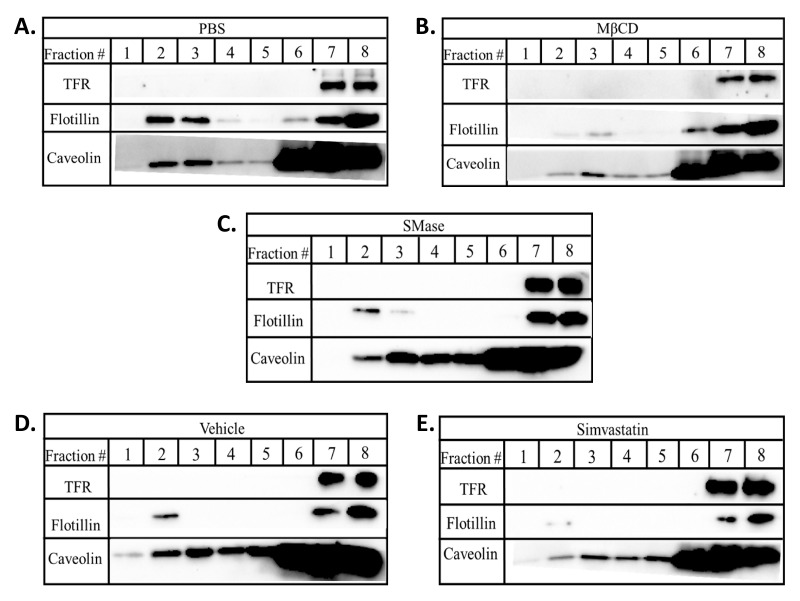
Lipid rafts disruption alters the levels of caveolin and flotillin in lipid raft fractions. BAECs were treated with (**A**) PBS; 2 h, (**B**) 10 mM MβCD; 2 h, (**C**) 0.25 U/mL SMase; 2 h, (**D**) vehicle; 3 h, or (**E**) 0.2 mM simvastatin; 2 h. Post treatment, cells were lysed and subjected to sucrose gradient centrifugation. Eight 500 µL fractions were collected from the top (fraction #1) to the bottom (fraction #8). Samples were subjected to SDS-PAGE and Western blotting.

Simvastatin treatment reduced VEGFR2 levels but did not affect GM-1 levels, thus, we explored other approaches to probe how this drug might influence lipid rafts. Lipid rafts were isolated in cold MNE buffer containing 1% Triton X-100 and subjected to discontinuous sucrose gradient ultracentrifugation. In control cells, the raft markers flotillin and caveolin were observed within the 5 and 25% sucrose gradient interface represented by fractions 2 and 3 while the non-raft marker TFR was only present in later more dense fractions 7 and 8 (Figure 4A). MβCD reduced the relative levels of flotillin and caveolin in raft fractions (Figure 4B). SMase reduced the levels of flotillin in raft fractions yet had little effect on caveolin distribution (Figure 4C). Lower relative levels of flotillin and caveolin in raft fractions were observed in simvastatin treated cells compared to the vehicle control treated cells (Figure 4D, E). Our data suggest that all three agents impact the lipids rafts leading to a reduction in caveolin and flotillin in raft fractions without affecting their overall expression. Furthermore, the relative differences in how the three agents affect GM-1, caveolin and flotillin suggest that each employs a somewhat distinct mechanism to disrupt rafts, yet all have in common the ability to reduce VEGFR2 levels.

### 2.3. Disruption of Lipid Rafts Increases Lysosomal Degradation of VEGFR2

**Figure 5:**
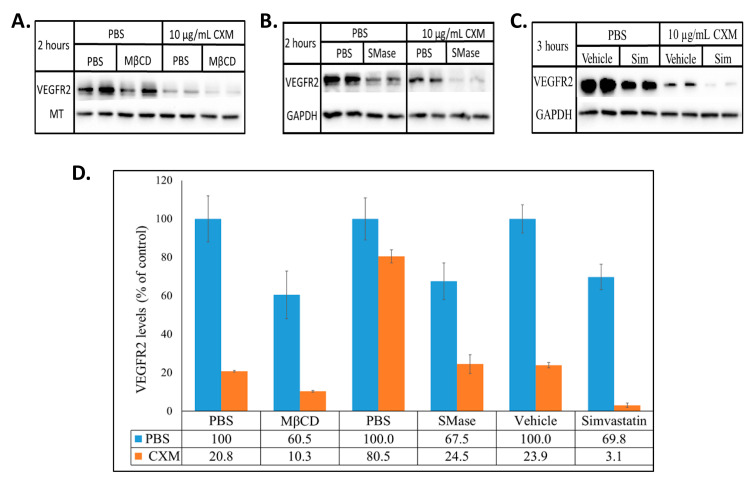
Raft disrupting agents reduce VEGFR2 levels in the absence of protein synthesis. BAECs were treated with (**A**) 10 mM MβCD ± 10 µg/mL CXM, (**B**) 0.25 U/mL SMase ± 10 µg/mL CXM, or (**C**) 0.2 mM simvastatin ± 10 µg/mL CXM for the stated time. Equal quantities of proteins from the whole cell lysates were subjected to SDS-PAGE and Western blotting. The optical density of VEGFR2, MT and GAPDH were densitometrically quantified with ImageJ. VEGFR2 was normalized to the respective house-keeping protein (MT or GAPDH) and the averaged percentage of VEGFR2 ±SD relative to its control (PBS or vehicle set to 100%) is presented (**D**).

The decrease in VEGFR2 expression in cells treated with lipid raft disrupting agents (Figure 1) indicates that lipid rafts are involved in maintaining the stability of VEGFR2. Disrupted lipid rafts may reduce VEGFR2 levels by either stimulating its degradation, reducing its synthesis or by affecting both processes. To evaluate these possibilities, cells were simultaneously treated with lipid raft disrupting agents and the protein synthesis inhibitor, cycloheximide (CXM), after which the VEGFR2 levels were quantified. There was approximately 40 and 80% reduction in VEGFR2 when the cells were treated with either MβCD alone or CXM alone. A combination of both CXM and MβCD caused a greater reduction in VEGFR2 levels by approximately 90% (Figure 5A,D). A similar trend was seen with the other two agents. Treatment of cells with either SMase alone or CXM alone caused approximately 33 and 20% reduction in VEGFR2 levels, respectively. A combination of both agents resulted in approximately 76% reduction in VEGFR2 levels (Figure 5B,D). Treatment with simvastatin alone, CXM alone or a combination of both caused a 30, 76 and 97% reduction in VEGFR2 levels, respectively (Figure 5C,D). The fact that raft disruption led to a decrease in VEGFR2 even in the absence of protein synthesis (CXM treatment), indicates that the reduction was reflective of increased VEGFR2 degradation.

**Figure 6:**
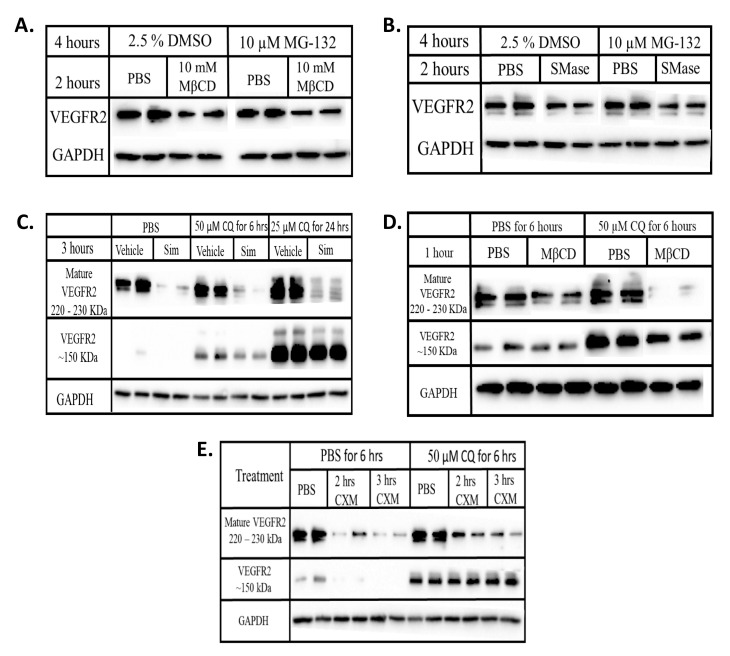
Disrupting lipid rafts increases the lysosomal degradation of VEGFR2. BAECs were treated with (**A**) MβCD ± MG-132 (**B**) SMase ± MG-132 (**C**) simvastatin ± CQ (**D**) MβCD ± CQ (**E**) CXM ± CQ for the indicated time duration. Equal quantities of proteins from each whole cell lysates were subjected to Western blotting as described in chapter 2. Chemiluminescence imaging was done on blots from the same gel and with the same exposure time. Post-imaging, MG-132 treatment blots were re-arranged for a better comparison.

VEGFR2 has been shown to be degraded by both the highly specific proteasome and the acidic lysosome. In fact, a crosstalk between both degradative pathways have been suggested especially when one machinery appears to be inadequate [22,24,32]. We observed that VEGFR2 levels were decreased when lipid rafts disruption was combined with the inhibition of protein synthesis (Figure 5) suggesting increased degradation. Inhibition of proteasomal degradation with MG-132 did not change VEGFR2 levels when compared to the DMSO treated control cells in the presence or absence of raft disruption (Figure 6A,B). This suggests the lack of involvement of the proteasome in the increased VEGFR2 degradation that is observed after raft disruption.

To explore the involvement of the lysosome, cells were simultaneously treated with a lipid raft disrupting agent and chloroquine diphosphate (CQ), a lysosomal inhibitor. CQ with or without simvastatin or MβCD did not restore the levels of mature VEGFR2 but instead led to the appearance of a ~150 kDa fragment of VEGFR2 (Figure 6C,D). CQ treatment in the presence of CXM also caused the appearance of this 150 kDa VEGFR2 (Figure 6E) suggesting that this low MW form does not represent newly synthesized immature VEGFR2. As a result, our data suggest that lysosomal inhibition prevents the complete degradation of VEGFR2, indicating that the increased degradation observed after raft disruption reflects lysosomal degradation.

### 2.4. Disrupting Lipid Rafts Decreases VEGF Induced ERK Activation

**Figure 7:**
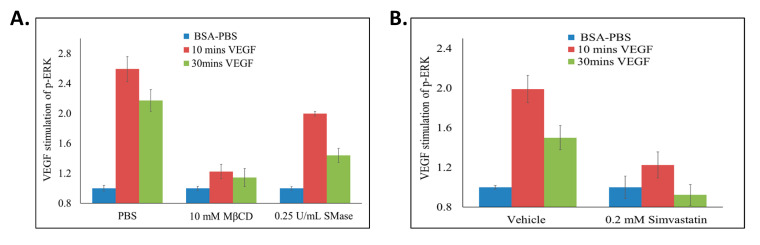
Disrupting lipid rafts decreases VEGF induced ERK activation. BAECs were seeded in 96 well cell culture dishes, serum starved and treated with (**A**) MβCD (10 mM; 2 h), SMase (0.25 U/mL; 2 h), or (**B**) simvastatin (0.2 mM; 3 h). During the final 10 or 30 min of lipid raft disruption, 25 ng/mL of VEGF was added to the cells. ELISA was conducted and total and phospho-ERK was quantified by measuring absorbance values (A450) above background (A570 nm). To calculate the percentage of VEGF induced ERK activation, p-ERK/ERK for all treatments were calculated. All values were then standardized to their respective unstimulated (1 mg/mL BSA-PBS) controls. The values obtained were used to calculate ERK activation as: %ERK Activation = (((treatment + VEGF) − (treatment))/((control + VEGF) − (control))) × 100. Averages ±SD of quadruplicate determinations are presented.

Endothelial cell proliferation and thus, the growth of healthy blood vessels require the stimulation of ERK/MAPK pathway by VEGF activation of VEGFR2 [3]. We sought to evaluate the consequence of the VEGFR2 degradation and down regulation that is associated with disrupting lipid rafts on VEGF activation of ERK. In the presence of MβCD, VEGF activated ERK by only 14 and 12% of what it did in the absence of MβCD after 10 and 30 min stimulation, respectively. A similar trend was observed in the presence of SMase, where VEGF activated ERK by 62 and 37%, respectively, of what it did in the absence of SMase (Figure 7A). VEGF treatment of cells in the presence of simvastatin for 10 min activated ERK by 23% of that observed in the absence of simvastatin, whereas VEGF treatment of simvastatin treated cells for 30 min showed no ERK activation (Figure 7B). Thus, the decreased levels of VEGFR2 caused by disrupting lipid rafts correlated with a reduction in VEGF-VEGFR2 induced ERK activation. This result suggests the importance of lipid rafts in regulating VEGFR2 dependent signaling pathways that modulate angiogenesis.

## 3. Discussion

**Figure 8:**
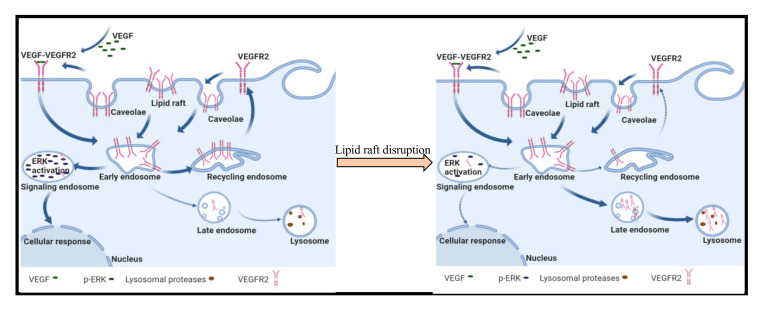
Model for lipid raft control of VEGFR2 trafficking in endothelial cells. Non-activated VEGFR2 is constitutively undergoing endocytosis and recycling to the plasma membrane where it can be activated by VEGF. Activated VEGF-VEGFR2 complexes are targeted to signaling endosomes leading to activation of ERK. In the presence of raft disrupting agents, we propose that VEGFR2 that is endocytosed is targeted to lysosomes for degradation leading to a reduction in steady state levels of the receptor on the plasma membrane, and consequently, reduced VEGF induced activation of ERK signaling.

While the role of lipid rafts in modulating cell surface receptor signaling has been an area of active research, there has been less focus on how lipid rafts impact the lifetime of resident proteins. In this study, we report that raft association of VEGFR2 modulates steady state levels of this receptor. We observed that disrupting lipid rafts with MβCD, SMase and simvastatin all significantly reduced VEGFR2 levels in endothelial cells (Figure 1). The effect on VEGFR2 appears to be relatively specific as the levels of caveolin and flotillin, two well characterized lipid raft associated proteins, were not affected by the raft disrupting agents (Figure 2). The selective reduction in VEGFR2 expression was correlated with increased VEGFR2 degradation and reduced VEGFR2 signaling of the ERK pathway after raft disruption. These findings suggest that modulation of lipid rafts may be a means to regulate endothelial responsiveness to VEGF by modulating steady state levels of VEGFR2 (Figure 8).

The study of VEGF activation of VEGFR2 has led to the development of FDA approved therapeutics such as the anti-VEGF molecules (bevacizumab and aflibercept) and tyrosine kinase inhibitors (sorafenib and sunitinib) for the treatment of certain cancers and wet AMD [33,34]. Unfortunately, these drugs, either as monotherapies or combination therapies, show only modest effectiveness in certain clinical settings, and are often beset by unwanted side effects [35,36]. Surprisingly, the use of simvastatin either alone or in combination with a chemotherapeutic has been shown to improve therapeutic outcomes and decrease mortality in patients with breast, prostate, lung and colorectal cancers [17,18]. The ability of statins to contribute to cancer therapy has been suggested to reflect an ability to modulate angiogenesis. Indeed, statins have been shown to have a biphasic effect whereby they promote angiogenesis at low doses (<0.1 µM) and inhibit angiogenesis at high doses (>1 µM) [37,38]. In the present study, we discovered that simvastatin induces the down regulation of VEGFR2, which may be part of the mechanism underlying its anti-angiogenic activity.

The three raft disrupting reagents used in this study each act via a distinct mechanism. MβCD and SMase both statistically decreased the levels of GM-1, a raft resident glycosphingolipid while simvastatin did not (Figure 2, Figure 3 and Table 1). This suggests that MβCD and SMase may disrupt lipid rafts by destabilizing all raft structures while simvastatin may influence a subclass of lipid rafts or might selectively impact raft formation such that its effects do not result in an overall change in GM-1 expression. The isopycnic isolation and subsequent characterization of lipid rafts showed reduced levels of the raft markers flotillin and caveolin in the buoyant, light density, raft fractions and indicating that all three agents disrupted raft structures. High doses of statins have been shown to impede VEGFR2 mediated signal transduction and inhibit the formation of blood vessels [39,40]. In fact, high doses of cerivastatin were shown to decrease VEGFR2 in hypoxic cells [41]. Our findings show that irrespective of the mechanism of action, all three agents targeted lipid rafts to decrease the expression of VEGFR2, which would be predicted to modulate VEGF-responsiveness and angiogenesis.

A regulated balance between synthesis and degradation is required for protein homeostasis. Treatment of cells with CXM, a general inhibitor of de novo protein synthesis, led to the expected decreased in the steady state levels of VEGFR2 as constitutive degradation was allowed to proceed (Figure 5A–C). Interestingly, when lipid rafts were disrupted in the presence of CXM, further reduction in the levels of VEGFR2 were observed (Figure 5) demonstrating that the raft disrupting agents caused an increase in VEGFR2 degradation. While an increase in VEGFR2 degradation is consistent with the decrease in steady state levels of VEGFR2, our results do not rule out the possibility that raft disrupting agents may also impact VEGFR2 synthesis.

Non-activated VEGFR2 undergoes constitutive endocytosis that leads to either recycling to the plasma membrane [5,42] or cellular proteolysis [43,44]. Degradative machineries that utilize ubiquination, have been implicated in the regulation of VEGFR2 levels [24,32,45]. However, inhibiting proteasomal degradation with MG-132 alone and in the presence of raft disrupting agents had no effect of VEGFR2 levels (Figure 6A,B). Lysosomal inhibition led to the appearance of a ~150 kDa fragment of VEGFR2 when cells were treated with either CQ alone or in combination with simvastatin or MβCD (Figure 6). The ~150 kDa VEGFR2 fragments were absent in cells subjected to CXM treatment but concurrent treatment with both CXM and CQ revealed a pronounced increase in its levels (Figure 6E). This indicates that the ~150 kDa VEGFR2 fragment is a derivative of existing VEGFR2 and does not represent a newly synthesize immature form of the receptor. Lysosomal inhibition of VEGF stimulated cells has been shown to reduce the levels of mature VEGFR2 while increasing the accumulation of the lower molecular weight VEGFR2 [21] indicating that this modified form of VEGFR2 in not subject to further proteolytic degradation in the lysosome. Taken together, our data suggest that lipid raft disruption decreases the levels of VEGFR2 by trafficking them to the lysosome for degradation. Previous findings have indicated that approximately 60% of VEGFR2 is within endosomal compartments at any time in order to protect the receptor from ectodomain shedding [5]. Lipid rafts may be playing a regulatory role by stabilizing VEGFR2 either on the plasma membrane or within selected endosomal compartments. Although the exact mechanism remains unknown, this process may involve the activity or certain ubiquinating enzymes, an activity yet to be characterized.

VEGF treatment of raft disrupted cells showed significantly reduced ERK activation when compared to control cells treated with VEGF only (Figure 7). This was not surprising as the increased degradation of VEGFR2 caused by disrupting lipid rafts leads to reduced levels of VEGFR2, which would be expected to translate to reduced activation of VEGFR2 and subsequently, reduced ERK activation. VEGF-VEGFR2 induces ERK activation through a series of phosphorylation events as part of the mechanism by which VEGF stimulates endothelial cell proliferation, differentiation and migration [46]. Thus, our study shows that lipid rafts influence non-activated VEGFR2 levels and the angiogenic signals it mediates.

## 4. Materials and Methods

### 4.1. Materials

Dulbecco’s Modified Eagle Media (DMEM), Phosphate Buffered Saline without calcium and magnesium (PBS), Penincillin/Streptomycin solution (P/S), L-Glutamine and 0.05% Trypsin/0.53 mM EDTA without sodium bicarbonate solution were purchased from Corning life sciences (Tewksbury, MA, USA). Methyl-β-Cyclodextrin (MβCD), sphingomyelinase (SMase), simvastatin, fluorescein isothiocyanate conjugated cholera toxin B subunit (CTB-FITC), chloroquine diphosphate and cycloheximide were purchased from Sigma Aldrich (Milwaukee, WI, USA). Caveolin polyclonal antibody (pAb) and MG-132 ready-made solution were purchased from Sigma Aldrich (Saint Louis, MO, USA). Newborn calf serum was purchased from Atlanta Biologics (Atlanta, GA, USA). Recombinant human VEGF_165_ protein (# 293-VE-010) was purchased from R & D systems (Minneapolis, MN, USA). VEGFR2 mAb (clone 55B11), ERK (clone 137F5), p-ERK (Thr202/Tyr204) GAPDH mAb (clone 14C10) and beta-tubulin (MT) pAb were purchased from Cell Signaling Technology (Danvers, MA, USA). Purified Mouse Anti-Flotillin-1 (clone 18/Flotillin-1) and FITC conjugated Goat Anti-Mouse IgG/IgM were purchased from Beckson Dickinson & Co (Franklin Lakes, NJ, USA). Transferrin Receptor (TFR) mAb (clone H68.4), EDTA-free protease and phosphatase inhibitor cocktail, TrypLE ^TM^ Express, Bicinchonic acid (BCA) protein assay kit, Bovine Serum Albumin Standard Pre-Diluted Set, and DAPI were purchased from Life technologies (Carlsbad, CA, USA). Goat anti-rabbit IgG conjugated to Horseradish peroxidase was purchased from Jackson ImmunoResearch (West Grove, PA, USA). Western Enhanced chemiluminescence (ECL) substrate was purchased from Bio-Rad (Hercules, CA, USA). TMB microwell peroxidase substrate system was purchased from KPL (Gaithersburg, MD, USA). Human Umbilical Vascular Endothelial Cells (HUVECs) pooled from donors and EGM™-2 Endothelial Cell Growth Medium-2 kit were purchased from Lonza (Walkersvile, MD, USA).

### 4.2. Cell Culture

Bovine Aortic Endothelial Cells (BAECs) were cultured and maintained in complete media (DMEM supplemented with 10% calf serum, 1X P/S, 1x L-Glutamine) at 37 °C and 5% pCO_2_. Serum starvation media is similar to complete media but has 0.1% calf serum. BAECs within passages 3–10 was used for all experiments. HUVECs in EGM^TM^-2 endothelial cell growth medium were cultured, maintained and used according to the manufacturer’s instructions. Starvation media was similar to EGM^TM^-2 endothelial cell growth medium but contained 0.1% fetal bovine serum and lacked VEGF. 

### 4.3. Simvastatin Activation

A slightly modified version of the procedure suggested by the manufacturer was used to activate simvastatin. A total of 650 µL of absolute ethanol and 937.5 µL of 0.1 N NaOH were added to 25 mg of the simvastatin powder and vortexed. The resulting solution was incubated at 50 °C for 2 h after which the pH was adjusted to 7.0 and then stored at 4 °C. The final concentration of activated simvastatin was 16 mg/mL or 38 mM. A vehicle solution consisting of the activation reagents and steps without simvastatin was also made. All lipid rafts disrupting agents dilutions were made in sterile PBS. VEGF dilutions were made in sterile 0.1% BSA-PBS.

### 4.4. Western Blot Analysis

BAECs at 160,000 cells/well in complete media were plated in 6 well tissue culture plates and incubated overnight at 37 °C and 5% pCO_2_. The cells were serum starved for 24 h after which they were treated with MβCD, SMase, simvastatin, CXM, MG-132 or CQ at concentrations and times specified in the figures and legends. After treatment, the cells were washed with cold PBS and lysed on ice with cold whole cell lysis buffer (1% triton X-100, 0.15 M NaCl, 0.01 M Tris pH 7.5, 1 mM EDTA pH 7.5, 1 mM EGTA pH 9.0, 0.5% NP-40) containing 1X protease and phosphatase inhibitor cocktail. Total protein concentration was determined using the BCA Protein Assay Kit according to manufacturer’s guide. Equal amounts of protein were loaded onto 7.5% polyacrylamide gels and subjected to SDS-PAGE. The separated proteins were transferred to a nitrocellulose membrane in Towbin’s transfer buffer (192 mM glycine, 25 mM tris, 20% methanol) at 100 V for 1 h. The membranes were blocked with 5% dried, non-fat milk in TTBS (10 mM Tris HCl + 0.1% tween 20) for 1 h at rt. Membranes were incubated overnight in blocking buffer containing primary antibodies against VEGFR2, caveolin, flotillin, TFR, GAPDH or MT (as indicated in the figures). The membranes were washed three times at room temperature with TTBS and incubated for 1 h at rt in appropriate HRP conjugated secondary antibodies in blocking buffer. Membranes were developed with the western ECL substrate and imaged using the ChemiDoc XRS+ System (Bio-Rad Life Science, Hercules, CA).

HUVECs at 200,000 cells/well in EGMTM-2 endothelial cell growth medium were plated in 6 well tissue culture plates and incubated overnight at 37 °C and pCO_2_ of 5%. The cells were serum starved for 24 h and treated with either 10 mM MβCD for 1 h or 0.2 mM simvastatin for 2 h. Cell lysis, SDS-PAGE and Western blotting were done as described above.

### 4.5. Image Flow Cytometry

BAECs (1.5 × 10^6^ cells) were seeded into 100 mm cell culture dishes in complete media and incubated for 24 h. The cells were serum starved for an additional 24 h and then treated with one of the following: PBS for 2 h, 10 mM MβCD for 2 h, 0.25 Units/mL SMase for 2 h, Vehicle for 3 h or 0.2 mM Simvastatin for 3 h. The cells were detached with TrypLE™ express. 1 × 10^6^ cells were collected by centrifugation and fixed with cold 3.7% formaldehyde in PBS at 4 °C for 15 min, permeabilized in 0.1% triton X-100 in PBS at room temperature for 10 min and blocked with cold 3% BSA-PBS on ice for 1 h. Lipid rafts were labeled with 5 µg/mL CTB-FITC in cold 3% BSA-PBS for 1 h on ice after and cells were counterstained with 1 µg/mL DAPI at rt for 5 min. The labeled cells were washed with cold PBS and suspended in 50 µL of cold 1% BSA-PBS. A minimum of 5000 single cells (aspect ratio >0.6; area >100) were acquired using the Amnis® FlowSight® Imaging Flow Cytometer and the INSPIRE software. The laser intensities were optimized for the labeled untreated cells and set constant across the treated cells. The acquired cell images were analyzed with the Amnis® IDEAS software. During the analysis, a template was created using the labeled untreated cells in which the maximum intensity from FITC-isotype control labeled cells was set as the background fluorescence. This template was applied across the treated cells for accurate comparison of CTB-FITC intensities.

### 4.6. Sucrose Gradient Ultracentrifugation

BAECs in complete media were plated at 500,000 cells/dish in 60 mm cell culture plates and incubated overnight at 37 °C and 5% pCO_2_. The cells were serum starved and treated with raft disrupting agents as indicated in the figure legend. The cells were washed with cold PBS and lysed on ice at 4 °C with freshly prepared MNE (25 mM MES, 150 mM NaCl, 2 mM EDTA pH 7.5) containing 1% Triton X-100 and 1X protease and phosphatase inhibitor cocktail. The lysates were centrifuged at 10,000× *g* at 4 °C for 5 min to remove cellular debris. A total of 600 µL of each supernatant was mixed with 600 µL of 80% sucrose solution made in double deionized water. One mL of the resulting lysate in 40% sucrose was placed at the bottom of a pre-chilled ultracentrifugation tube. A discontinuous gradient was made in which 2 mL of 30% sucrose was layered over the lysate followed by 1 mL of 5% sucrose solution. Samples were subjected to ultracentrifugation at 240,000× *g* for 18 h at 4 °C. Post ultracentrifugation, 8 fractions of 500 µL volumes were collected from the top to the bottom of each tube. 48 µL of each fraction was mixed with 12 µL of sample buffer containing DDT, boiled for 5 minutes, and subjected to SDS-PAGE and Western blotting as previously described.

### 4.7. Enzyme Linked Immunosorbent Assay

BAECs were seeded at a density of 4000 cells/200 µL of complete media in each well of a 96 well tissue culture dish and incubated for 24 h at 37 °C and 5% pCO_2_. The cells were serum starved and treated with raft disrupting agents. In the final 10 and 30 min of treatment, the cells were treated with 25 ng/mL VEGF_165_. Cells were washed once on ice with cold PBS, incubated in 95% methanol for 10 min, and fixed in 4% paraformaldehyde for 20 min on ice. Cells were washed 4× with cold PBS and blocked overnight at 4 °C with 3% BSA in Tris-buffered saline (50 mM Tris HCl pH 7.4, 0.15 M NaCl) on the shaker. The following day, the cells were washed once with TBS and incubated with either anti-ERK (1:1000) or anti-p-ERK (1:1000) in blocking buffer for 1.5 h at rt. The cells were incubated with goat anti-rabbit HRP secondary antibody washed 3× with 0.1% Tween 20 in TBS (TTBS) followed by 3× with TBS. TMB substrate (100 µL) was added to each well (10 min) and the reaction stopped with 100 µL of 0.1 N phosphoric acid. The absorbance of the resulting solution was read at 450 nm with an additional background reading at 570 nm.

## 5. Conclusions

The current successful use of statins in the management of certain cancers raises the question of how they influence angiogenesis. Our data shows that disrupting the highly organized structure of lipid rafts increases the degradation of VEGFR2 and consequently decreases VEGF-mediated activation of ERK. Statins have been in clinical use for years with manageable side effects that are not as severe as those of the current anti-angiogenic therapies. Our data provide insight into how the relatively safe simvastatin may also be beneficial in the management of tumor growth and cancers by specifically regulating the levels of the angiogenic VEGFR2 and subsequently reducing VEGF mediated signaling in endothelial cells.

## Figures and Tables

**Table 1:** Statistical parameters of cells populations with perturbed lipid rafts ^1^.

	Fluorescent Intensities (RFU) of CTB-FITC Positive Cells					
Treatment	Minimum	Median	Maximum	Mean	% GM-1 left	% GM-1 depleted
PBS	23,224.79	156,234.35	804,216.16	180,238.41		
MβCD	23,175.07	31,574.43	210,109.05	34,916.51	19.4	80.6
SMase	23,319.45	49,614.78	166,897.55	54,143.37	30.0	70.0
Vehicle	17,394.01	97,560.02	387,299.64	103,439.69		
Simvastatin	15,123.00	97,678.44	382,942.39	104,230.59	98.9	1.1

^1^ The minimum, maximum, mean and median fluorescent intensities values from Figure 3A,B are represented in the table. The % GM-1 ganglioside remaining and depleted were calculated as: %GM-1 remaining = (MFI of treatment/MFI of control) × 100; %GM-1 depleted = 100 − %GM-1 remaining.

## Abbreviations

BAEC	Bovine Aortic Endothelial Cells
CTB	Cholera Toxin B Subunit
CXM	Cyclohexemide
ERK	Extracellular-signal Regulated Kinases
GM-1	Monosialotetrahexosylganglioside
HUVECs	Human Umbilical Vascular Endothelial Cells
MAPK	Mitogen Activated Protein Kinases
MβCD	Methyl-β-Cyclodextrin
RME	Receptor Mediated Endocytosis
SMase	Sphingomyelinase
TBS	Tris Buffered Saline
TFR	Transferrin Receptor
VEGF	Vascular Endothelial Growth Factor
VEGFR	Vascular Endothelial Growth Factor Receptor
